# Pigmented poroma on the scalp clinically mimicking basal cell carcinoma^☆^^☆☆^

**DOI:** 10.1016/j.abd.2020.08.028

**Published:** 2021-09-24

**Authors:** Masato Ishikawa, Mikio Ohtsuka, Toshiyuki Yamamoto

**Affiliations:** Department of Dermatology, Fukushima Medical University, Fukushima, Japan

A 73-year-old Japanese woman visited our department complaining of a nodule on the scalp which had appeared four years previously. Physical examination revealed a 12-mm semi-pedunculated black nodule on the left side of the head (Fig. 1). Dermoscopic examination showed large blue-gray ovoid nest-like structures, irregularly dilated vessels, and erosions. Histopathological examination showed a nodular tumor extending from the epidermis into the mid-dermis (Fig. 2). The tumor was composed of small round cells that had a high nucleocytoplasmic ratio, with small pores, which are features of sweat duct differentiation features of poroid differentiation into small ductal structures (Fig. 3). There were no histopathological features suggestive of basal cell carcinoma (BCC). Some of the tumor cells contained melanin granules, and an increased number of melanocytes, confirmed by HMB-45 staining and MART-1 staining, was observed within the nests. Also, many melanophages were observed in the stroma. After making a diagnosis by punch biopsy, the nodule was removed under local anesthesia.

**Figure 1:**
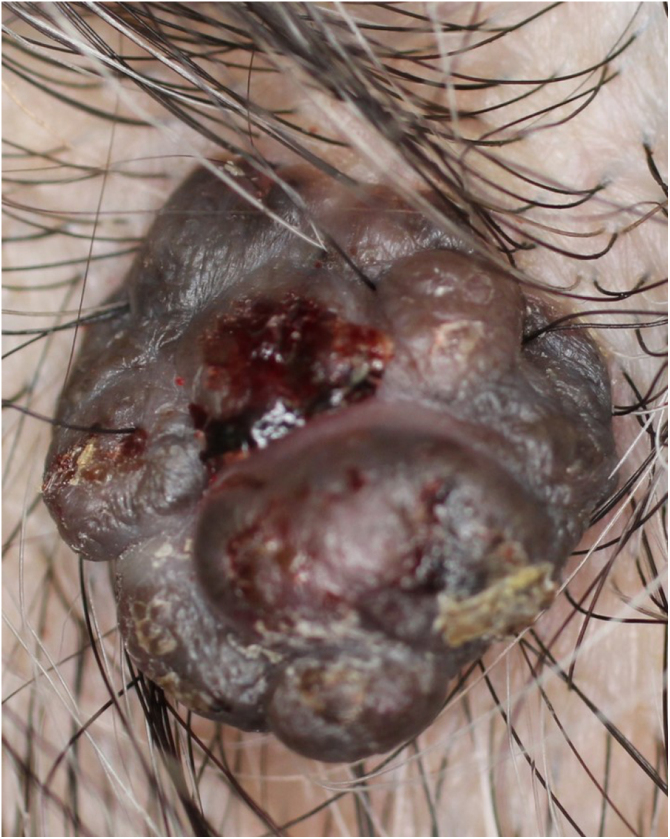
A clinical appearance of semi-pedunculated black nodule on the left side of the head.

**Figure 2:**
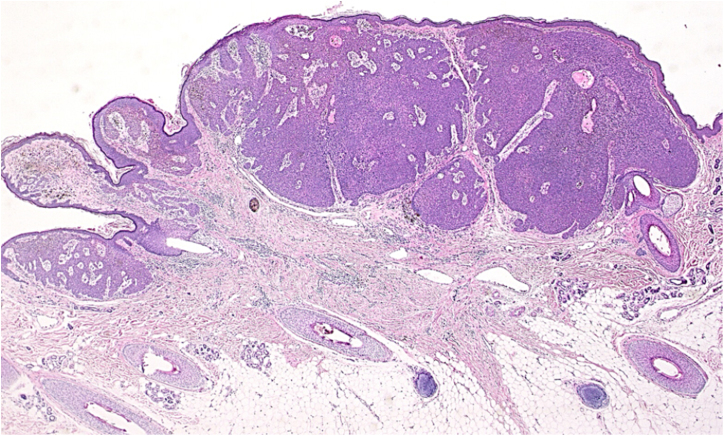
Histopathological examination of the lesion revealed a nodular tumor extending from the epidermis into the mid-dermis (Hematoxylin-eosin stain, original magnification, 20×).

**Figure 3:**
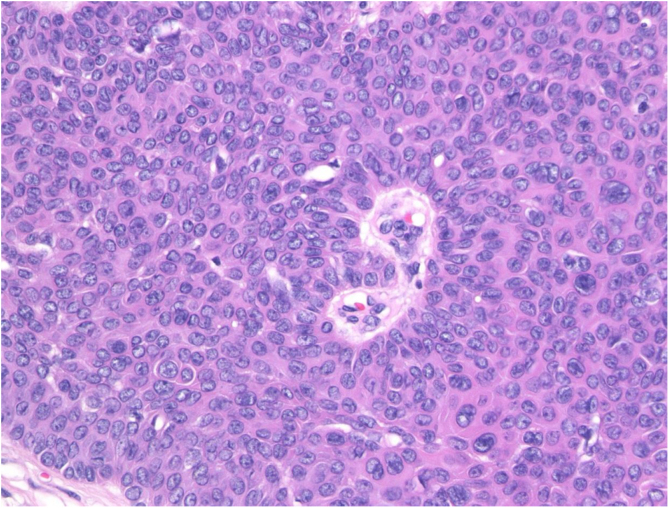
Detail of the histopathological examination: The tumor was composed of small round cells which had a high nucleocytoplasmic ratio, with small pores, which are features of sweat duct differentiations (Hematoxylin-eosin stain, original magnification, 100×).

Eccrine poroma is a benign adnexal tumor mainly composed of poroid cells and often present as a reddish nodule. While eccrine poroma does not appear to have a bias for occurrence between races, pigmented variants of eccrine poroma often develop in non-white races. Pigmented eccrine poroma have been reported especially from Japan, and according to a clinicopathological analysis in Japan, among the 421 cases with pathological diagnosis of poroid cell neoplasms, 114 cases (27.1%) had melanin pigment in the tumor cells.^1^, ^2^, ^3^

As shown in the present case, pigmented eccrine poroma on the scalp can clinically mimic BCC. Previous studies have shown that pigmented eccrine poroma has dermoscopic findings of arborizing vessels and blue-gray ovoid nests, and pigmented eccrine poroma on the face was clinically similar to BCC.^4^ In the present case, the gross pathology are similar to those of BCC. In addition, since there were no dermoscopic findings of seborrheic keratosis or malignant melanoma, and the scalp is one of the most frequent areas where BCC occurs, the lesion was suspected to be BCC until the biopsy.

Minagawa and Koga found in their case series study that the most frequent dermoscopic structures in pigmented eccrine poromas were vascular structures such as arborizing vessels, hairpin vessels, and polymorphous vessels.^2^ However, the dermoscopic characteristics of other skin tumors such as globule-like structures and comedo-like openings were also found in pigmented eccrine poromas.^2^ One possible reason why pigmented eccrine poroma shows similar dermoscopic findings to BCC and/or seborrheic keratosis is that, as both of these tumors are classified into appendage tumors, their rough structures are similar, and they are distinguished only by pathological findings that cannot be observed by dermoscopy. Although Bombonato et al. suggested that reflectance confocal microscopy may be useful for diagnosing pigmented eccrine poroma, biopsy is still essential for the diagnosis in order to avoid misdiagnosis and overtreatment.^5^ In conclusion, considering the lack of established specific dermoscopic criteria for pigmented eccrine poroma, pigmented eccrine poroma on the scalp should be biopsied for histopathologic confirmation of the diagnosis.

## Financial support

None.

## Authors’ contributions

Masato Ishikawa: Designed the study; performed the research and contributed to analysis and interpretation of data; wrote the initial draft of the manuscript; read and approved the final version of the manuscript.

Mikio Ohtsuka: Performed the research and contributed to analysis and interpretation of data; read and approved the final version of the manuscript.

Toshiyuki Yamamoto: Designed the study; assisted in the preparation of the manuscript; read and approved the final version of the manuscript.

## Conflicts of interest

None declared.

## References

[1] Kuo H.W., Ohara K. (2003). Pigmented eccrine poroma: a report of two cases and study with dermatoscopy. Dermatol Surg.

[2] Minagawa A., Koga H. (2010). Dermoscopy of pigmented poromas. Dermatology.

[3] Ito K., Ansai S., Kimura T. (2009). A clinicopathological analysis of 421 cases of poroid cell neoplasms 4th report: Histopathological subfindings. J Dermatol.

[4] Kassuga L.E., Jeunon T., Sousa M.A., Campos-do-Carmo G. (2012). Pigmented poroma with unusual location and dermatoscopic features. Dermatol Pract Concept.

[5] Bombonato C., Piana S., Moscarella E., Lallas A., Argenziano G., Longo C. (2016). Pigmented eccrine poroma: dermoscopic and confocal features. Dermatol Pract Concept.

